# Clinical considerations for the treatment of patients with familial chylomicronemia syndrome using a hepatic-targeted *APOC3* antisense oligonucleotide

**DOI:** 10.1016/j.ajpc.2025.101352

**Published:** 2025-11-16

**Authors:** Archna Bajaj, Elif A. Oral, Alan Brown, Daniel Gaudet, Veronica J. Alexander, Ewa Karwatowska-Prokopczuk, Seth J. Baum

**Affiliations:** a Division of Translational Medicine and Human Genetics, University of Pennsylvania, Philadelphia, PA 19104, USA; b Division of Metabolism, Endocrinology and Diabetes (MEND), Department of Internal Medicine, Michigan Medicine, University of Michigan, Ann Arbor, MI 48109, USA; c Division of Cardiology, Advocate Heart Institute at Advocate Lutheran General Hospital, Park Ridge, IL 60068, USA; d Department of Medicine, Université de Montréal and ECOGENE-21, Chicoutimi, QC, Canada; e Ionis, Carlsbad, CA 92010, USA; f Department of Integrated Medical Sciences, Charles E. Schmidt College of Medicine, Florida Atlantic University, Boca Raton, FL 33431, USA; Flourish Research, Boca Raton, FL 33434, USA

**Keywords:** Familial chylomicronemia syndrome, Hypertriglyceridemia, Olezarsen, Antisense oligonucleotide, Apolipoprotein C-III, Acute pancreatitis

## Abstract

Familial chylomicronemia syndrome (FCS) is a rare, typically debilitating genetic disorder of extreme hypertriglyceridemia associated with high triglyceride levels and elevated risk for recurrent acute pancreatitis. Diagnosis of FCS is frequently delayed due to its rarity, and treatment options are limited. Patients often report history of acute pancreatitis or associated symptoms, including chronic or recurrent abdominal pain, weakness, and fatigue. The hallmark of chylomicronemia (extreme hypertriglyceridemia) syndromes, including FCS, is extremely high triglyceride levels ≥880 mg/dL (10 mmol/L) resistant to conventional triglyceride-lowering medications including statins, fibrates, and omega-3 fatty acids. Validated clinical scoring tools or genetic testing can support diagnosis. Patients must follow a strict FCS-specific diet <15 to 20 g fat/day. Failure to adhere increases the possibility of recurrent acute and chronic pancreatitis and pancreatic dysfunction. Dietary adherence and long-term disease management are extremely challenging for patients. Multidisciplinary clinical teams can improve patient outcomes and quality of life. Therapies that reduce apolipoprotein C-III, a regulator of triglyceride metabolism, offer an FCS treatment option. Olezarsen, a hepatic-targeted *APOC3* antisense oligonucleotide, is the first US Food and Drug Administration–approved therapy specifically for FCS treatment, indicated as an adjunct to diet to reduce triglycerides in adult patients with FCS; olezarsen is also European Medicines Agency–approved. Combining olezarsen with the low-fat FCS diet may prevent acute pancreatitis and improve long-term patient outcomes. Plozasiran, a small interfering RNA therapy targeting *APOC3*, is in late-stage clinical development. This article describes the updated diagnosis and clinical management of FCS and practical considerations for *APOC3*-targeting treatment.

## Graphical abstract

**Figure:**
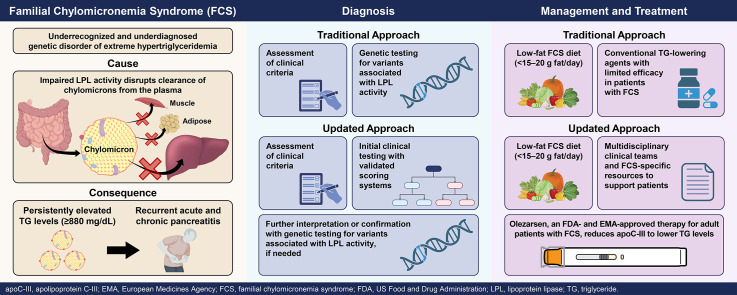

## 1 Introduction

Familial chylomicronemia syndrome (FCS) is a rare genetic disorder of extreme hypertriglyceridemia (HTG) associated with persistent chylomicronemia and recurrent, potentially fatal acute pancreatitis episodes [[1], [2], [3], [4]]. The prevalence of FCS is thought to be up to 13 per 1,000,000 people in the US and up to 19 per 1,000,000 people globally [[5], [6], [7]] but is likely underestimated because FCS is rare and complex to identify [[8], [9], [10]]. The underlying pathogenic mechanism is inadequate lipoprotein lipase (LPL) activity impairing the clearance of chylomicrons (large, triglyceride [TG]-rich lipoproteins [TRLs] produced after a meal) from the plasma [4]. Chylomicronemia increases the risk for HTG-induced acute pancreatitis [1,11] that is more severe than non–HTG-induced acute pancreatitis [4,8].

Timely recognition is essential for effective FCS management and treatment. Two validated clinical scoring tools, along with genetic testing, are now available to diagnose FCS [4,10,12]. FCS management is evolving, but a low-fat FCS-specific diet (<15–20 g fat/day) remains essential [3,13,14]. Multidisciplinary teams including specialist clinicians and registered dieticians are key in supporting patients’ dietary adherence and optimizing long-term disease management to prevent severe complications like acute pancreatitis [1,3,4]. Newly available FCS support programs and resources can further support physicians in helping patients manage dietary restrictions and symptoms.

Most patients with FCS do not respond to conventional TG-lowering therapies such as statins, fibrates, and omega-3 fatty acids. However, in recent clinical trials, *APOC3*-targeted oligonucleotide therapies reduce TG levels and appear to decrease acute pancreatitis incidence in patients with FCS [1,11,[15], [16], [17], [18]]. Volanesorsen, an antisense oligonucleotide (ASO) designed to target *APOC3* messenger RNA (mRNA) in patients with FCS [16,19,20], is approved in the EU, Great Britain, Brazil, and Chile, but not the US [17]. It is indicated as an adjunct to diet in patients with genetically identified FCS and at high risk for pancreatitis who have not responded adequately to diet and conventional TG-lowering therapy [[21], [22], [23], [24]]. Subsequent research enabled the development of olezarsen, a hepatocyte-targeted triantennary *N*-acetylgalactosamine (GalNAc)–conjugated ASO approved by the US Food and Drug Administration (FDA) in 2024 as an adjunct to diet to reduce TG levels in adult patients with FCS [17,25] and by the European Medicines Agency in 2025 as an adjunct to diet in adult patients for the treatment of genetically confirmed FCS [26]. Plozasiran, an *APOC3*-targeted small interfering RNA (siRNA) conjugated to triantennary GalNAc, is in late-stage clinical development for FCS treatment [18,27].

This review provides updates on the identification, diagnosis, and management of patients with FCS in the era of *APOC3*-targeted therapies, emphasizing clinical considerations for olezarsen use.

## 2 Identification of FCS

### 2.1 Clinical presentation

**Fig. 1:**
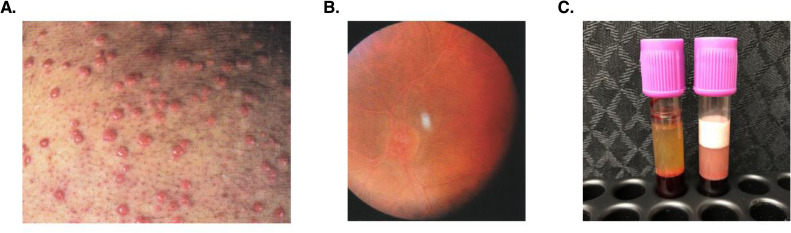
Clinical manifestations of FCS. (A) Eruptive cutaneous xanthomas, (B) lipemia retinalis, and (C) plasma sample from an FCS patient compared with a control healthy individual after overnight storage at 4 °C [28]. Figure reprinted with permission from: Baass A, et al, Familial Chylomicronemia Syndrome: An Under-Recognized Cause of Severe Hypertriglyceridaemia. 2020;287(4):340-348. John Wiley & Sons, Inc. Copyright © 1999–2025 John Wiley & Sons, Inc. or related companies. All rights reserved, including rights for text and data mining and training of artificial intelligence technologies or similar technologies. FCS, familial chylomicronemia syndrome.

Patients with extreme HTG, including FCS, may develop eruptive xanthomas (small, yellowish dermatological papules with an erythematous base, 3–5 mm diameter; Fig. 1a). Lipemia retinalis (whitened retinal vessels without vision impairment) may be apparent during ophthalmologic examination (Fig. 1b) [1,28,29]. Blood sampling often reveals lactescent (milky) serum due to the presence of chylomicrons [13,28]. After overnight storage of serum at 4 °C, chylomicrons form a creamy supernatant layer (Fig. 1c) [28]. Patients with FCS may report generalized abdominal pain, bloating, physical weakness, indigestion, fatigue, and cognitive symptoms such as difficulty concentrating, impaired judgment, and brain fog [30]. Physical symptoms may present at any age from childhood to adulthood [4,31]. The majority of patients have a history of acute pancreatitis [32,33] that may have necessitated hospitalization and/or resulted in long-term complications [1]. Pancreatitis recurs in ∼50% of patients [33,34], and many patients report daily abdominal pain between episodes [35].

The laboratory hallmark of FCS is extremely high circulating TG levels ≥880 mg/dL (≥10 mmol/L) [11,31]. Other characteristics include TG/total cholesterol (TC) ratio >8 (measured in mg/dL) and apolipoprotein B (apoB) levels <100 mg/dL (1.0 g/L) [10,36]. ApoB can help distinguish FCS from other forms of persistent HTG like multifactorial chylomicronemia syndrome (MCS), in which apoB levels may overlap with the FCS range but are generally higher (≥75 mg/dL) [2,29,36]. Patients with FCS typically lack characteristics associated with secondary or multifactorial HTG, such as obesity, metabolic syndrome, high alcohol consumption, untreated hypothyroidism, chronic renal insufficiency, pregnancy, or uncontrolled type 2 diabetes [8,28,29,37]. Although these conditions when present do not exclude FCS as the cause of HTG, their absence raises suspicion for FCS. Another feature suggestive of FCS is a lack of response (TG levels decrease by <20%) to conventional TG-lowering medications, including statins, fibrates, and omega-3 fatty acids [1,11,31,38]. A summary of 66 patients with genetically identified FCS from the Balance trial, a phase 3 study of olezarsen, provides a sample of characteristics found in adults with FCS (Table 1) [17].

**Table 1:** Patient demographics and baseline characteristics of 66 patients with genetically identified FCS in Balance, a phase 3, double-blind, placebo-controlled clinical trial of olezarsen [17].

	Placebo	Olezarsen 50 mg	Olezarsen 80 mg
	(*n* = 23)	(*n* = 21)	(*n* = 22)
Demographics			
Age, years, mean (SD)	44.0 (14.7)	43.2 (12.1)	47.7 (13.3)
Sex, n (%)			
Female	12 (52.2)	15 (71.4)	11 (50.0)
Male	11 (47.8)	6 (28.6)	11 (50.0)
Race/ethnicity, n (%)			
White	22 (95.7)	17 (81.0)	17 (77.3)
Hispanic or Latino	3 (13.0)	3 (14.3)	1 (4.5)
Asian	0	3 (14.3)	3 (13.6)
Native Hawaiian or Pacific Islander	0	1 (4.8)	0
Other	1 (4.3)	0	2 (9.1)
Medical history			
History of acute pancreatitis in prior 10 years, n (%)	15 (65.2)	15 (71.4)	17 (77.3)
Episodes per patient in prior 10 years, mean (SD)*	6.6 (16.5)	4.1 (4.4)	4.8 (7.5)
Hypertension, n (%)	6 (26.1)	3 (14.3)	4 (18.2)
Tobacco user, n (%)	0	3 (14.3)	2 (9.1)
Type 1 or 2 diabetes mellitus, n (%)	6 (26.1)	3 (14.3)	7 (31.8)
Previous treatment with volanesorsen, n (%)	10 (43.5)	8 (38.1)	8 (36.4)
Baseline thrombocytopenia, n (%)^†^	4 (17.4)	4 (19.0)	2 (9.1)
Physical exam findings			
Weight, kg, mean (SD)	67.8 (16.1)	61.2 (11.6)	68.4 (16.7)
BMI, kg/m^2^, mean (SD)	24.2 (4.1)	22.4 (3.5)	25.1 (6.0)
Laboratory test results			
Triglycerides, mg/dL			
Mean (SD)	2596 (1256)	2684 (1235)	2613 (1499)
Median (range)	2493 (334, 5436)	2679 (779, 5965)	2086 (683, 6898)
ApoC-III, mg/dL, mean (SD)	27.7 (11.7)	27.7 (10.5)	27.5 (11.6)
Total cholesterol, mg/dL, mean (SD)	286.0 (113.9)	323.4 (100.5)	277.4 (99.3)
LDL cholesterol, mg/dL, mean (SD)	16.7 (8.4)	17.6 (8.5)	22.8 (14.1)
HDL cholesterol, mg/dL, mean (SD)	14.7 (3.8)	15.7 (4.0)	14.5 (4.5)
Total apoB, mg/dL, mean (SD)	59.7 (18.9)	65.2 (13.5)	58.4 (17.2)
ApoB-48, mg/dL, mean (SD)	14.2 (14.2)	18.5 (15.3)	11.6 (8.1)
Chylomicron triglyceride, mg/dL, mean (SD)	2269 (1237)	2302 (1265)^‡^	2477 (2123)
Chylomicron-*C* + VLDL cholesterol,^§^ mg/dL, mean (SD)	255.3 (114.4)	293.2 (103.1)	245.7 (110.9)
Non-HDL cholesterol, mg/dL, mean (SD)	271.3 (113.3)	307.6 (101.8)	262.9 (100.4)
ALT, U/L, mean (SD)	23.5 (16.6)	19.3 (12.6)	23.2 (16.9)
AST, U/L, mean (SD)	23.4 (6.8)	21.4 (9.7)^‡^	25.6 (12.2)
eGFR, mL/min/1.73 m^2^, mean (SD)	109.7 (19.4)	111.3 (19.0)	105.5 (18.9)
Platelets 10^9^/L, mean (SD)	214.8 (72.0)	200.1 (61.88)	188.2 (58.36)
UACR, g/mol, mean (SD)	2.68 (3.78)	1.86 (3.03)	7.11 (14.64)
UPCR, g/mol, mean (SD)	10.7 (7.11)	11.0 (6.86)	19.4 (36.82)
Concomitant medications, n (%)			
Statin	7 (30.4)	4 (19.0)	5 (22.7)
Omega-3 fatty acid	7 (30.4)	6 (28.6)	12 (54.5)
Fibrate	11 (47.8)	8 (38.1)	11 (50.0)
Other lipid-lowering agent	3 (13.0)	0	3 (13.6)
Antidiabetic	6 (26.1)	3 (14.3)	8 (36.4)
Antihypertensive	7 (30.4)	2 (9.5)	7 (31.8)
Oral anticoagulants	9 (39.1)	1 (4.8)	3 (13.6)
Tamoxifen, estrogen, or progestin	0	6 (28.6)	0

From *New England Journal of Medicine*, Stroes ESG, et al., Olezarsen, Acute Pancreatitis, and Familial Chylomicronemia Syndrome, 390, 1781-1792, Copyright © 2024 Massachusetts Medical Society. Reprinted with permission from Massachusetts Medical Society.

^⁎^Historical pancreatitis events were obtained from the medical record and not adjudicated if >5 years old. One patient in the placebo group had a history of 79 acute pancreatitis events. Without this patient, the mean (SD) number of acute pancreatitis episodes in the prior 10 years in the placebo group is 3.3 (4.9).

^†^Thrombocytopenia was defined as a platelet count lower than 140,000 per cubic millimeter.

^‡^*n* = 20.

^§^This variable represents the cholesterol content in the density fraction of <1.006 g per milliliter and represents the combined cholesterol content of the chylomicron and VLDL fractions.

ALT, alanine aminotransferase; apoB, apolipoprotein B; apoB-48, apolipoprotein B-48; apoC-III, apolipoprotein C-III; AST, aspartate aminotransferase; BMI, body mass index; eGFR, estimated glomerular filtration rate; FCS, familial chylomicronemia syndrome; HDL, high-density lipoprotein; LDL, low-density lipoprotein; SD, standard deviation; UACR, urine albumin to creatinine ratio; UPCR, urine protein to creatinine ratio; VLDL, very-low-density lipoprotein.

### 2.2 Diagnosis

FCS has traditionally been diagnosed by genetic testing or clinical criteria [4,9,17]. Genetic identification is based on homozygosity, compound heterozygosity, or double heterozygosity for known pathogenic variants in causative genes (*LPL, APOC2, APOA5, GPIHBP1*, or *LMF1*) [9]. Genetic testing can confirm a clinical diagnosis or clarify an ambiguous clinical profile. Interpreting genetic testing results is complex. A result of “indeterminate,” a common finding [10], does not exclude FCS because while most patients with FCS have ≥2 documented loss-of-function variants in LPL machinery, not all possible pathogenic variants are characterized [2,10]. Therefore, clinicians may consider initial clinical testing with validated clinical scoring systems to differentiate FCS from other forms of extreme HTG [4,10].

The European FCS Expert Panel developed a scoring system in 2018 to differentiate between FCS and MCS based on fasting plasma TG levels, secondary HTG factors, history of pancreatitis, presence of abdominal pain, history of familial combined hyperlipidemia, response to treatment for hyperlipidemia, and age of symptom onset (**Table S1**) [11]. A score of ≥10 indicates FCS is very likely, ≤9 means FCS is unlikely, and ≤8 means FCS is very unlikely [11]. This score was validated in a large, ethnically and genetically diverse cohort of patients with FCS or MCS in the UK [12]. Other clinical scoring systems are published but not validated [39,40].

The North American FCS (NAFCS) score was recently developed by expert physicians to identify FCS [10]. Scoring criteria include age, age of HTG onset, body mass index (BMI), history of pancreatitis, presence of secondary factors, and laboratory values (TG >880 mg/dL, TG/TC ratio >8 [measured in mg/dL], and apoB <100 mg/dL [<1.0 g/L]; **Table S2**) [10]. This score was validated in a population of patients with FCS or MCS [10] and showed a strong concordance with positive genetic diagnosis in patients from the Balance study of olezarsen [41]. A score of ≥60 indicates “definite FCS,” 45 to 60 is consistent with “likely FCS,” and 30 to 44 should prompt further genetic testing [10].

If FCS is suspected based on clinical criteria or genetic testing, clinicians should consider referring the patient to a lipidologist or other specialist with experience treating FCS [3]. Dieticians familiar with FCS are also critical for optimally reducing TG levels.

## 3 Traditional management and treatment of FCS

### 3.1 Diet

Regardless of adjunct pharmacologic treatment, patients with FCS must adhere to a strict, very low-fat FCS diet (<15–20 g fat/day) and restrict simple carbohydrates and alcohol, which can increase TG levels and trigger acute pancreatitis [3,4,14]. Fat-soluble vitamins (A, D, E, K) should be monitored and possibly supplemented in the FCS diet [14]. Purified medium-chain triglycerides (MCTs) do not promote chylomicron synthesis and may be a good source of fat [8,32]; while MCT oils are commercially available, they are potentially costly.

Standard “low-fat” recipes for patients with hypercholesterolemia or other cardiometabolic conditions may still be too high in fat for a true FCS diet, in which limiting fat intake to <20 g/day is recommended. Resources with low-fat recipes are available to support adherence to the FCS diet (**Table S3**). Specialized dietary programs that are personalized for the patient and developed in collaboration with a registered dietician can help develop sustainable eating habits. The clinical team should counsel patients to be careful during special events, and meal planning in advance can facilitate dietary adherence [14]. Patients with FCS must follow a low-fat diet indefinitely, and clinical teams should reevaluate the dietary plan after starting medications to determine whether further restriction of fat intake is needed to achieve target TG levels.

Patients with FCS may develop type 3c diabetes due to insulin insufficiency resulting from acute pancreatitis [42]. They may also have concurrent type 2 diabetes, although this is not typical [17,42,43]. Many diabetes diets have unsuitably high fat content for patients with FCS. Diabetes educators and dieticians should be aware of FCS and the need for low dietary fat (<15–20 g/day) to provide appropriate counseling.

### 3.2 Medications and comorbid conditions

Common TG-lowering medications (eg, statins, fibrates, and omega-3 fatty acids) are unlikely to help patients with FCS [1,4,14,38]. Omega-3 fatty acids also increase overall fat intake [14], but docosahexaenoic acid and eicosapentaenoic acid may be supplemented at very low doses because they cannot be synthesized in sufficient amounts by the human body. Some clinicians may choose to withhold omega-3 fatty acids in patients with TG levels exceeding 800 mg/dL until TG levels fall below 500 mg/dL through diet and/or medications. Medications that may increase TG levels (listed comprehensively in the NAFCS scoring system [10]) should be avoided. Comorbidities that raise TG levels, such as hypothyroidism and uncontrolled diabetes, should be investigated and treated [10,11].

## 4 Apolipoprotein C-III

**Fig. 2:**
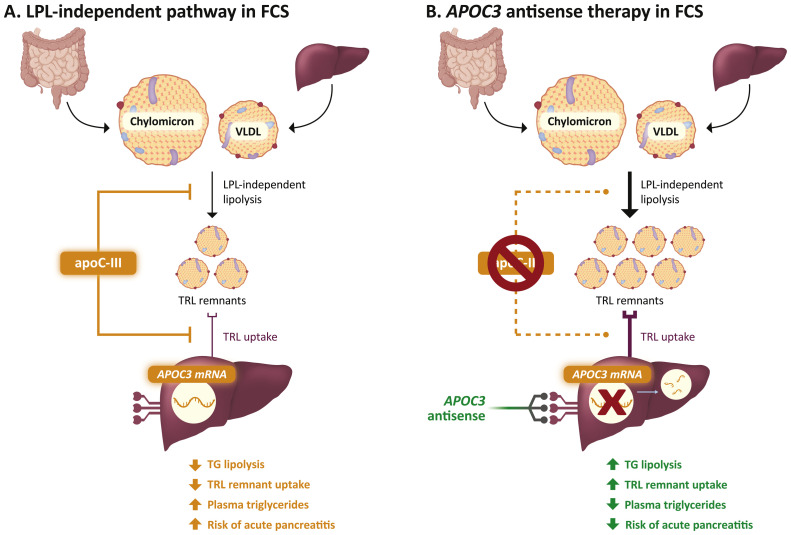
LPL-independent pathway in FCS and treatment with *APOC3* antisense therapy. *APOC3*, apolipoprotein C3; apoC-III, apolipoprotein C-III; FCS, familial chylomicronemia syndrome; LPL, lipoprotein lipase; TG, triglyceride; TRL, TG-rich lipoprotein; VLDL, very-low-density lipoprotein.

Recent therapeutic approaches to FCS focus on apolipoprotein C-III (apoC-III), a 79-amino acid glycoprotein encoded by the *APOC3* gene [4]. Initial studies on loss-of-function *APOC3* variants revealed a correlation between apoC-III and TG levels, highlighting *APOC3* as a promising therapeutic target for TG reduction [44,45]. The presumed primary mechanism by which apoC-III raises TG levels is inhibition of LPL-mediated lipolysis [46], implying that apoC-III reduction would be ineffective in patients with FCS and inadequate LPL activity [19]. However, evidence of an apoC-III–mediated pathway for LPL-independent hepatic clearance of TRLs [[47], [48], [49], [50]] prompted clinical studies demonstrating that apoC-III reduction via *APOC3* RNA-directed therapies (volanesorsen, olezarsen, and plozasiran) can indeed lower TG levels in patients with FCS [[16], [17], [18], [19]], although the effect may be stronger in clinically identified patients with residual LPL activity relative to those with genetically identified FCS [51]. These agents are not disease-modifying and do not address the cause of FCS; however, they improve lipid clearance, likely by enhancing LPL-independent hepatic uptake of TRLs (Fig. 2) [19,52]. As olezarsen is now available in the US, its development, clinical data, and practical considerations for use are summarized as follows.

## 5 Treatment with olezarsen

### 5.1 Development of olezarsen

Volanesorsen is an earlier ASO designed to reduce *APOC3* mRNA that was approved in the EU in 2019 as an adjunct to diet in adult patients with genetically identified FCS and at high risk for pancreatitis [21]. Volanesorsen treatment in 3 patients with FCS initially confirmed that apoC-III reduction could lower TG levels in patients with FCS who lack adequate LPL activity [19]; volanesorsen also appeared to reduce pancreatitis incidence in the subsequent APPROACH trial, a phase 3 placebo-controlled study in 66 patients with FCS [16,53]. Volanesorsen was not approved in the US due to concerns about thrombocytopenia [16,17]. Advancements in conjugating ASOs to highly tissue-specific ligands provided a potential mechanism for avoiding off-target reductions in platelet count observed with volanesorsen treatment. Olezarsen differs from volanesorsen by the inclusion of 3 GalNAc residues that bind to asialoglycoprotein receptors on hepatocytes, permitting ASO delivery to hepatocyte nuclei where *APOC3* mRNA is generated and allowing for greater potency and less systemic exposure [16,17,54]. Otherwise, olezarsen has the same nucleotide sequence, chemical composition, and mechanism of action as volanesorsen [17]. Olezarsen was approved in the US in December 2024 at a recommended dosage of 80 mg administered as a subcutaneous injection once monthly as an adjunct to diet to reduce TG levels in adults with FCS; the US indication does not require a genetic diagnosis [25]. In Europe, olezarsen was approved in September 2025 as an adjunct to diet in adults for the treatment of genetically confirmed FCS [26].

### 5.2 Clinical efficacy of olezarsen

**Fig. 3:**
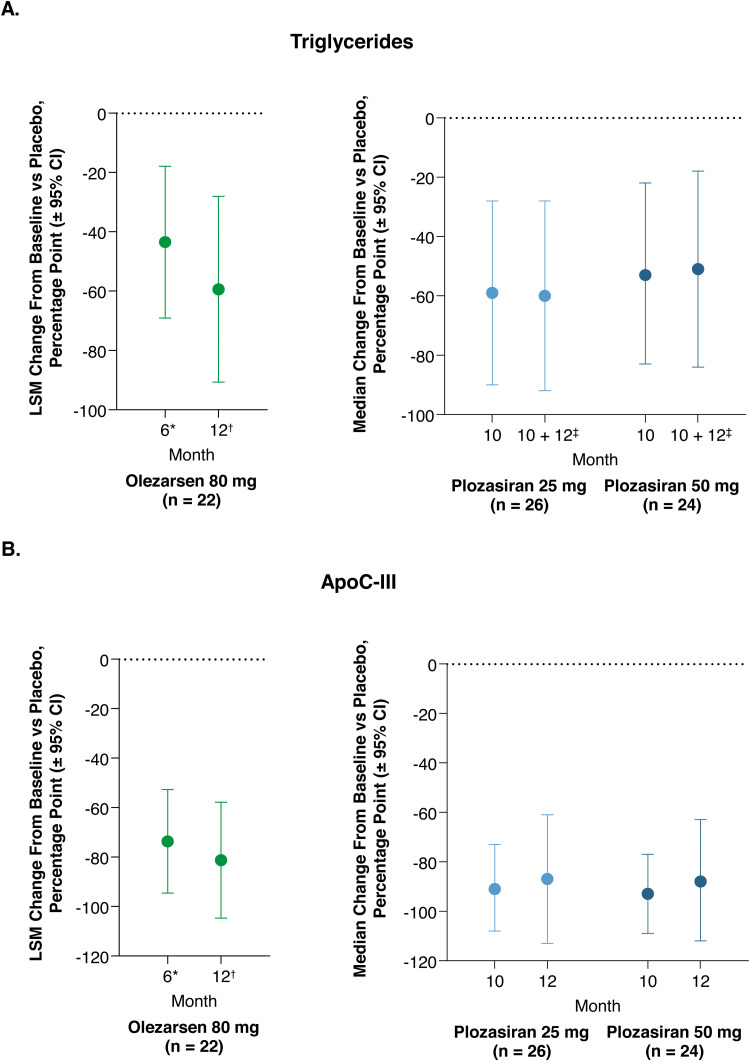
Reduction in fasting plasma levels of triglycerides (A) and apoC-III (B) with olezarsen and plozasiran [17,18].

The phase 3 Balance trial assessed the efficacy and safety of olezarsen administered subcutaneously every 4 weeks for 53 weeks in 66 patients with genetically identified FCS (Table 1) [17]. The trial met the primary endpoint of reduction from baseline in TG levels following olezarsen 80 mg vs placebo after 6 months [17]; olezarsen 80 mg was also associated with apparent reductions from baseline in apoC-III (months 6 and 12), TG levels (month 12; Fig. 3) [17], and acute pancreatitis incidence (1 and 11 acute pancreatitis episodes among 22 and 23 patients receiving olezarsen 80 mg and placebo, respectively), although these were not statistically significant under the prespecified testing hierarchy [17]. Patients were counseled to maintain an FCS diet during the study [17].

*Endpoint calculated from an average of weeks 23, 25, and 27. ^†^Endpoint calculated from an average of weeks 51 and 53. ^‡^Endpoint calculated as the mean of months 10 and 12. apoC-III, apolipoprotein C-III; CI, confidence interval; LSM, least-squares mean.

### 5.3 Clinical safety of olezarsen

It is unknown whether adverse reactions in clinical practice will reflect those observed in trials with olezarsen, as long-term real-world data are not yet available. Clinicians should monitor for adverse reactions described in the FDA-approved prescribing information that occurred in >5% of olezarsen-treated patients (either dose) and at >3% higher frequency than in placebo-treated patients in Balance, which included injection-site reactions (19% vs 9%), decreased platelet count (12% vs 4%), and arthralgia (9% vs 0%) [25].

Per the prescribing information, the most common reason for treatment discontinuation was hypersensitivity reactions, defined as symptoms of bronchospasm, diffuse erythema, facial swelling, urticaria, chills, or myalgia [25]. Notably, these treatment discontinuations were not attributed to hypersensitivity in the Balance study publication due to differing clinical and regulatory definitions. The adverse events (AEs) that led to discontinuation in 3 patients treated with olezarsen (2, olezarsen 80 mg; 1, olezarsen 50 mg) were reported as diarrhea, vomiting, chest discomfort, chills, myalgia, trismus, flushing, and 1 death (olezarsen 50 mg; considered unrelated to treatment) [17]. Since the FDA identified chills and myalgia as hypersensitivity reactions, some events captured as hypersensitivity reactions in the prescribing information were likely reported in Balance as influenza-like illness, defined as AEs of influenza-like illness, pyrexia, feeling hot, body temperature increased, chills, myalgia, or arthralgia, starting on the day of injection or the next day [17]. One patient in each olezarsen group and no placebo-treated patients experienced influenza-like illness [17]. More familiar hypersensitivity reactions such as urticaria were not associated with treatment discontinuation in Balance: no patients experienced an anaphylactic reaction according to the narrow FDA Medical Query [17], and no reported hypersensitivity AEs, defined by the narrow Standardized MedDRA Query as the preferred terms of dermatitis, eczema, and macular rash, led to treatment discontinuation [17].

Since patients may not recognize influenza-like symptoms as a hypersensitivity reaction, clinicians should advise patients to read the FDA-approved patient labeling, inform them about the signs and symptoms of hypersensitivity reactions to olezarsen, and instruct them to seek medical attention and discontinue olezarsen if such reactions occur [25]. If a patient experiences an influenza-like illness but wishes to continue treatment, subsequent steps should be informed by symptom severity and shared clinician-patient decision-making.

### 5.4 Monitoring laboratory results during olezarsen treatment

The authors recommend regular laboratory testing in patients prescribed olezarsen to monitor efficacy and safety (Table 2). The European FCS Expert Panel recommends TG testing at least 4 times/year following pancreatitis in patients with unstable TG levels and at least twice a year in those with stable TG levels and no history of HTG-induced pancreatitis. Clinicians may consider monitoring TG levels and lipids more frequently during olezarsen treatment. If TG levels remain unchanged or increase, the patient’s diet should be investigated, and the fat content further reduced if warranted.

**Table 2:** Monitoring laboratory results during olezarsen treatment [11,17,25,65].

	Considerations	Author recommendations
Triglycerides	• A patient’s triglyceride level is expected to decrease during olezarsen treatment but may remain unchanged or even increase • To monitor the risk for pancreatitis, the European FCS Expert Panel recommends TG testing at least 4 times per year directly following pancreatitis in patients who have unstable TG levels and at least twice a year in patients who have stable TG levels without a history of HTG-induced pancreatitis	• Clinicians may consider monitoring the patient’s TG and lipid panels more frequently during olezarsen treatment • Perform a lipid panel every 3 months for the first year of olezarsen treatment, then every 6 months thereafter • Investigate the patient’s diet and lower fat content if triglyceride levels remain elevated or respond minimally with olezarsen treatment

Platelet count reduction	• In the Balance trial, the mean platelet count in patients receiving olezarsen 80 mg was 188,000/mm^3^ at baseline and decreased by 10% after a year of treatment • No patients reported a reduction in platelet count below 50,000/mm^3^ • No major bleeding events were associated with low platelet counts • The proportion of patients who experienced any bleeding adverse event was similar across olezarsen and placebo groups	• Perform a complete blood count every 6 months for the first year of olezarsen treatment, then every 12 months thereafter

Fasting glucose and HbA1c	• In the Balance trial, small increases in fasting glucose (≤17 mg/dL) and HbA1c (<0.2 percentage points) levels were observed in olezarsen-treated patients • Per the prescribing information, the incidence of hyperglycemia was higher in olezarsen-treated patients without a medical history of diabetes at baseline (52%) compared with those treated with placebo (35%)	• Monitor fasting blood glucose every 12 months; for patients with diabetes, monitor HbA1c levels every 12 months

Increases in liver enzyme levels	• In the Balance trial, increases in baseline liver enzyme levels in patients who received olezarsen were within the normal range, occurring throughout the first 3 months and stabilizing thereafter; liver enzyme levels returned towards baseline with discontinuation of olezarsen • No patient experienced hepatic failure; none of the olezarsen-treated patients had ALT levels ≥3 × ULN, only 1 olezarsen-treated patient had AST levels ≥3 × ULN, no patient had total bilirubin levels ≥2 × ULN, and none met the criteria for Hy’s Law	• Perform a liver function test at months 1 and 3, then every 6 months thereafter • Increase testing frequency if elevations or abnormalities are observed • No dose adjustments are recommended for patients with mild hepatic impairment; olezarsen has not been studied in patients with moderate or severe hepatic impairment • In the Balance study, the stopping rules for liver chemistry elevations specified that olezarsen would be stopped if: ALT or AST >8 × ULN (confirmed); ALT or AST >5 × ULN (confirmed) that persisted for ≥2 weeks; ALT or AST >3 × ULN (or the greater of 2 × baseline value or 3 × ULN if baseline was >ULN; confirmed) and total bilirubin >2 × ULN or INR >1.5; ALT or AST >3 × ULN (or the greater of 2 × baseline value or 3 × ULN if baseline was >ULN; confirmed) and the new appearance (i.e., onset coincides with the changes in hepatic enzymes) of fatigue, nausea, vomiting, right upper quadrant pain or tenderness, fever, rash, and/or concomitant eosinophilia (>ULN) [17]

LDL-cholesterol and apoB	• Olezarsen is expected to increase LDL cholesterol and apoB levels in patients with FCS, who characteristically have low levels of LDL cholesterol • In the Balance trial, mean LDL cholesterol and total apoB increased over 6 months in olezarsen-treated patients • The maximum LDL cholesterol value remained <70 mg/dL for 74% of olezarsen-treated patients • Increases in LDL cholesterol were accompanied by a decrease in total non-HDL cholesterol	• Perform a lipid panel every 3 months for the first year of olezarsen treatment, then every 6 months thereafter • The physician-authors recommend intensifying LDL–cholesterol-lowering therapies in patients who are at high ASCVD risk and whose LDL cholesterol level remains below target

Physician-authors: A Bajaj, EAO, A Brown, DG, and SJB.

ALT, alanine aminotransferase; apoB, apolipoprotein B; ASCVD, atherosclerotic cardiovascular disease; AST, aspartate aminotransferase; FCS, familial chylomicronemia syndrome; HbA1c, hemoglobin A1c; HDL, high-density lipoprotein; HTG, hypertriglyceridemia; INR, international normalized ratio; LDL, low-density lipoprotein; TG, triglyceride; ULN, upper limit of normal.

Olezarsen may lower platelet count [25]. In Balance, the mean baseline platelet count in patients receiving olezarsen 80 mg was 188,000/mm^3^ and decreased by a mean of 10% after a year of treatment [25]. No patients reported platelet count reduction <50,000/mm^3^, and there were no major bleeding events associated with low levels [25]. Fasting glucose and hemoglobin A1c (HbA1c) may increase with olezarsen. Small increases in fasting glucose (≤17 mg/dL) and HbA1c (<0.2 percentage points) occurred in olezarsen-treated patients in Balance [17,25]. Liver enzyme levels may also increase. In Balance, baseline liver enzyme levels increased within normal range during the first 3 months of olezarsen treatment and stabilized thereafter [25]. Liver enzyme levels returned towards baseline with discontinuation of olezarsen [25]. Lastly, patients with FCS typically have low levels of low-density lipoprotein cholesterol (LDL-C), and apoC-III reduction can increase LDL-C and apoB levels from baseline in this population. In Balance, patients treated with olezarsen had increased LDL-C and total apoB levels compared with those receiving placebo; 74% of olezarsen-treated patients maintained maximum LDL-C levels <70 mg/dL [25].

### 5.5 Olezarsen in special populations

Clinicians may have special concerns for patients with FCS who become pregnant, are children, or are older patients receiving multiple medications. Pregnancy is relevant because rising TG levels during the third trimester may be severely elevated in FCS and increase the risk for acute pancreatitis [25]. Olezarsen is not approved for use in pregnancy, and there are currently no data on olezarsen in pregnant women or its presence in the breast milk of lactating women, its effects on the breastfed infant, or its impact on milk production [25]. Pediatric patients were excluded from Balance and are ineligible for olezarsen [17,25]. No differences in the safety or effectiveness of olezarsen were observed in patients ≥65 years old (38% of patients in trials to date; *n* = 111) compared with younger adults [25]. Dose adjustment is not necessary for older patients [25] and drug-drug interactions are not anticipated [25].

Dose adjustment is also not required for patients with mild to moderate renal impairment (estimated glomerular filtration rate ≥30 to <90 mL/min) [25] or mild hepatic impairment [25]. Patients with more severe renal or hepatic impairment were excluded from Balance, limiting available information in these populations [25].

### 5.6 Olezarsen usage information

Comprehensive patient information and usage instructions for olezarsen are available in the prescribing information (**Fig. S1**) [25]. Patients receiving olezarsen should remain on an FCS diet (<20 g fat/day) to achieve expected treatment outcomes [25]. Alternating injection sites between administrations is recommended to reduce the possibility of injection-site reactions. Symptomatic interventions, such as icing the injection site prior to and after dosing, can be considered.

## 6 Management of acute pancreatitis

An episode of acute pancreatitis necessitates hospitalization with aggressive intravenous hydration, initial bowel rest, pain control, and careful monitoring for organ failure [55,56]. Patients should receive intravenous fluids while refraining from oral intake for ≥48 h, with gradual attempts to introduce clear liquids orally; after clear liquids for an additional 48 h, patients can gradually advance to soft and whole foods as tolerated while maintaining a total fat intake of <20 g/day. Although clinical guidelines recommend enteral tube feeding within 24 h over parenteral nutrition in patients experiencing acute pancreatitis [57,58], this may be more appropriate for non–HTG-induced pancreatitis and is not advised for patients with FCS.

Insulin infusions may stimulate LPL activity and lower TG levels in patients with insulin-deficient diabetes presenting with HTG due to insulin deficiency but are ineffective in those with complete LPL deficiency and can cause hypoglycemia [3,8,59,60]. Plasmapheresis, if available, can rapidly reduce TG levels and manage acute pancreatitis [59], but there is no convincing evidence to support lipoprotein (chylomicron) apheresis as an effective form of long-term treatment for extreme HTG [4,8]. Both intravenous insulin and plasmapheresis are not generally recommended for HTG-induced acute pancreatitis [60].

Patients with FCS presenting with acute pancreatitis may sometimes be misdiagnosed as HTG due to alcohol abuse or as drug-seeking behavior for reported chronic abdominal pain. Consultation with the patient’s FCS specialist at the time of presentation may be helpful for making the correct diagnosis.

## 7 Future FCS therapies

### 7.1 Plozasiran

Plozasiran, an siRNA that reduces hepatic apoC-III production, is in late-stage clinical development for FCS treatment [4,18]. In the phase 3 PALISADE trial of 75 patients with genetically identified FCS or symptomatic persistent chylomicronemia, plozasiran 25 mg and 50 mg administered subcutaneously every 3 months met the primary endpoint of reduction from baseline in TG levels at 10 months vs placebo; plozasiran was associated with significantly reduced apoC-III levels and TG levels compared with placebo (Fig. 3) [18]. When data from the plozasiran 25 and 50 mg groups were pooled, the treatment was associated with decreased acute pancreatitis incidence compared with pooled placebo [18]. AE frequency was generally similar between patients receiving plozasiran vs placebo; severe and serious AEs were more common in placebo-treated patients [18]. There were no deaths or AEs involving hypersensitivity or anaphylaxis. Mild injection-site reactions occurred in 1 (4%), 4 (15%), and 1 (4%) patient in the plozasiran 50 mg, 25 mg, and placebo groups, respectively [18]. HbA1c increased in 3 patients in each plozasiran group and in no placebo-treated patients [18]. No patients experienced alanine aminotransferase or aspartate aminotransferase levels >3 × the upper limit of normal, and platelet levels remained similar to baseline in all groups [18].

Olezarsen and plozasiran have not been directly compared in clinical trials. Comparisons between the two phase 3 trials are confounded by differences in the patient populations: Balance enrolled only patients with genetically identified FCS [17], while PALISADE enrolled patients with genetically identified FCS or symptomatic persistent chylomicronemia [18]. Additionally, the data in PALISADE and Balance were analyzed differently: the primary endpoint in Balance was the least-squares mean change in TG levels from baseline to month 6 [17], while the primary endpoint in PALISADE was the median change in TG levels from baseline to month 10 (Fig. 3) [18]. In January 2025, the FDA accepted the New Drug Application for plozasiran for the treatment of FCS, with a Prescription Drug User Fee Act action date in November 2025 [61].

### 7.2 Other APOC3-targeted therapies

RN0361 is a liver-directed, *APOC3*-targeted siRNA being investigated in a phase 1 study in healthy adult participants and a phase 2 study in adult patients with HTG (including patients with FCS; NCT06471543) [62]. CS-121 is an in vivo *APOC3* base-editing therapy delivered by lipid nanoparticles under investigation in a phase 1 study in adults with FCS (NCT07176923) [63]. Primary completion of the studies for both agents is anticipated in late 2026.

## 8 Limitations

*APOC3*-targeting therapies are approved [21,25,26] or under investigation [18,62,63] for only adult patients with FCS; therefore, there remains an unmet need for therapies for pediatric patients with FCS. There also remains a need for information on the use of these therapies during pregnancy; pregnancy tends to increase TG levels, and this increase may be severe in genetically susceptible patients [25,64]. Notably, the indications for both volanesorsen and olezarsen emphasize the use of these agents as adjuncts to diet [21,25,26], affirming that low-fat diet remains the mainstay of treatment for FCS regardless of the magnitude of TG lowering observed with *APOC3*-targeting therapies. The FDA prescribing information for plozasiran, anticipated in late 2025, will clarify whether the same dietary guidance will apply to the entire class—as with the Balance study [17], the PALISADE study required patients to maintain a low-fat diet [18]—and real-world evidence may provide some insight into the importance of consistent dietary adherence in combination with *APOC3*-targeting pharmacologic treatment over the long term. Additionally, the requirement for genetic testing differs between regulatory agencies; in the US, olezarsen may be prescribed to patients with FCS without genetic testing [25], while in Europe, a genetically confirmed diagnosis is required [26]. It remains to be seen whether these requirements will be consistently applied for plozasiran and what implications this may have regarding patient access.

## 9 Conclusions

Patients with FCS face persistent risk for acute pancreatitis and its complications despite a low-fat diet and conventional TG-lowering medications. Clinicians must be alerted to the signs and symptoms of FCS to ensure timely diagnosis and appropriate management. The recent approval of olezarsen in the US as an adjunct to diet in adult patients with FCS provides an opportunity to reduce TG levels and potentially lower the risk for acute pancreatitis.

## Funding

This work was supported by Ionis Pharmaceuticals, Inc.

## CRediT authorship contribution statement

**Archna Bajaj:** Writing – review & editing, Writing – original draft, Conceptualization. **Elif A. Oral:** Writing – review & editing, Writing – original draft, Conceptualization. **Alan Brown:** Writing – review & editing, Writing – original draft, Conceptualization. **Daniel Gaudet:** Writing – review & editing, Writing – original draft, Conceptualization. **Veronica J. Alexander:** Writing – review & editing, Writing – original draft, Conceptualization. **Ewa Karwatowska-Prokopczuk:** Writing – review & editing, Writing – original draft, Conceptualization. **Seth J. Baum:** Writing – review & editing, Writing – original draft, Conceptualization.

## Declaration of competing interest

The authors declare the following financial interests/personal relationships which may be considered as potential competing interests:

**A.Ba.** has received research support and/or consultation fees from Alexion Pharmaceuticals, Amgen, Arrowhead Pharmaceuticals, Chiesi, Eli Lilly, Ionis, Kaneka Corporation, NewAmsterdam Pharma, Novartis, and Regeneron Pharmaceuticals. **E.A.O.** has received clinical trial support, grant support, and/or consultation fees from Akcea Therapeutics/Ionis, Amryt Pharma (now part of Chiesi), Fractyl Health, Gemphire Therapeutics, GI Dynamics, Marea Therapeutics, Novo Nordisk, Regeneron Pharmaceuticals, and Rhythm Pharmaceuticals; served as an advisor to Akcea Therapeutics/Ionis, Amryt Pharma (now part of Chiesi), and Regeneron Pharmaceuticals; has an invention disclosure filed for novel mibavademab effects under review; has royalty rights from the use of metreleptin in lipodystrophy; and is a recipient of federal grants from the National Institute of Diabetes and Digestive and Kidney Diseases. **A.Br.** reports consultation fees from Amgen, Arrowhead Pharmaceuticals, Ionis, NewAmsterdam Pharma, Pfizer, and Regeneron Pharmaceuticals and received fees paid to their institution from Amgen, Arrowhead Pharmaceuticals, and Ionis. **D.G.** reports consultation fees, clinical trials fees, or research grants from Alnylam Pharmaceuticals, Amgen, Arrowhead Pharmaceuticals, AstraZeneca, Boehringer Ingelheim, Chiesi (Amryt Pharma), CRISPR Therapeutics, Dalcor Pharma, Eli Lilly, Esperion Therapeutics, Ionis, Kowa Pharmaceuticals, NewAmsterdam Pharma, Novartis, Novo Nordisk, Pfizer, Regeneron Pharmaceuticals, Sanofi, Ultragenyx, and Verve Therapeutics. **V.J.A.** is an employee of Ionis. **E.K.P.** was an employee of Ionis when this review was written. **S.J.B.** reports personal fees from Akcea Therapeutics/Ionis, Altimmune, Amgen, Axcella Health, AstraZeneca, Boehringer Ingelheim, Eli Lilly, Esperion Therapeutics, Madrigal Pharmaceuticals, Novartis, Regeneron Pharmaceuticals, and Sanofi.

## Glossary

AE: adverse event
*APOA5*: apolipoprotein A5
*APOC2*: apolipoprotein C2
*APOC3*: apolipoprotein C3
apoB: apolipoprotein B
apoC-III: apolipoprotein C-III
ASO: antisense oligonucleotide
BMI: body mass index
FCS: familial chylomicronemia syndrome
FDA: US Food and Drug Administration
GalNAc: *N*-acetylgalactosamine
*GPIHBP1*: glycosylphosphatidylinositol-anchored high-density lipoprotein binding protein 1
HbA1c: hemoglobin A1C
HTG: hypertriglyceridemia
LDL-C: low-density lipoprotein cholesterol
*LMF1*: lipase maturation factor 1
*LPL*: lipoprotein lipase
LPL: lipoprotein lipase
MCS: multifactorial chylomicronemia syndrome
MCT: medium-chain triglyceride
MedDRA: Medical Dictionary for Regulatory Activities
mRNA: messenger RNA
NAFCS: North American FCS
siRNA: small interfering RNA
RNA: ribonucleic acid
TC: total cholesterol
TG: triglyceride
TRL: TG-rich lipoprotein

## Appendix Supplementary materials

Image, application 1
