# Supplementary material for: Clinical considerations for the treatment of patients with familial chylomicronemia syndrome using a hepatic-targeted APOC3 antisense oligonucleotide
Source: Am J Prev Cardiol. 2025 Nov 16;24:101352. doi: 10.1016/j.ajpc.2025.101352 (PMC12686656; doi:10.1016/j.ajpc.2025.101352)
Supplement: Supplementary file 1 [file mmc1.pdf]

Supplementary Materials

Table S1. The European FCS Expert Panel score calculator.

Table S2. The North American FCS score calculator.

Table S3. Support resources for patients with FCS and their clinicians.

Fig. S1. General usage instructions for olezarsen.

Table S1. The European FCS Expert Panel score calculator [1].

Criteria	Score
**Fasting TG >10 mmol/L for 3 consecutive blood analyses***	5
Fasting TG >20 mmol/L at least once	1
**Previous TG <2 mmol/L**	–5
**No secondary HTG factors (except pregnancy† and ethinylestradiol)**	2
**History of pancreatitis**	1
**Unexplained recurrent abdominal pain**	1
**No history of familial combined hyperlipidemia**	1
**No response (TG decrease <20%) to hypolipidemic treatment**	1
**Onset of symptoms at age (years)**	
<40	1
<20	2
<10	3
**Diagnosis**	
FCS very likely	≥10
FCS unlikely	≤9
FCS very unlikely	≤8

*Plasma concentrations measured at least 1 month apart.

†If the diagnosis is made during pregnancy, a secondary assessment is necessary to confirm the diagnosis postpartum.

FCS, familial chylomicronemia syndrome; HTG, hypertriglyceridemia; TG, triglyceride.

Table S2. The North American FCS score calculator [2].

Criteria	Score
**Current age (years)**	
<1*	0
≥1–9	12
≥10	0
**HTG onset (years; used only if patient is ≥10 years at evaluation)**	
<10	12
≥10	0
**BMI (percentile for children/adolescents)**	
<25 kg/m^2 or <85th percentile	9
≥25 kg/m^2 or ≥85th percentile	0
**History of pancreatitis**	
Pancreatitis	16
Abdominal pain but no pancreatitis	9
Neither abdominal pain nor pancreatitis	0
**Presence of secondary HTG factors**	
None	11
≥1	0
**Laboratory values (select all that apply)**	
TG >880 mg/dL	13
TG/TC ratio >8[†]	8
apoB <100 mg/dL (1.0 g/L)[‡]	12
**Other criteria (select all that apply)**	
Patient ≥1–9 years old and has no secondary HTG factors	7
TG/TC >8[†] and apoB <100 mg/dL (1.0 g/L)[‡]	7
TG/TC >8[†] and has no secondary HTG factors	5
**Diagnosis**	
Definite FCS	≥60
Likely FCS	45–60
Further genetic testing is required to confirm potential FCS	30–44

Online versions of the NAFCS score calculator can be found at https://tgaware.com/diagnosing-fcs/fcs-diagnostic-scoring/ and https://www.lipid.org/nla/north-american-familial-chylomicronemia-calculator-or-nafcs-scoring-tool.

*The NAFCS score cannot be calculated for patients <1 year old. If an infant presents with no secondary factors that may contribute to HTG, consider a diagnosis of FCS. If an infant presents with ≥1 secondary factor that may contribute to HTG, but with 2 TG readings >880 mg/dL and unexplained failure to thrive, consider a diagnosis of FCS.

†When measured in mg/dL.

‡Measurement of apoB is potentially unavailable in routine clinical practice.

apoB, apolipoprotein B; BMI, body mass index; FCS, familial chylomicronemia syndrome; HTG, hypertriglyceridemia; NAFCS, North American FCS; TC, total cholesterol; TG, triglyceride.

Table S3. Support resources for patients with FCS and their clinicians.

Description	Website
**Diagnosis**	
**Foundation of the NLA: find a clinician**<br>Search engine for locating a healthcare provider specializing in lipid disorders, although they may not all be experienced with treating FCS.	https://www.learnyourlipids.com/find-a-clinician/
**FCS Foundation: genetic testing**<br>Telehealth and clinic-based no-cost genetic testing and counseling.	https://www.livingwithfcs.org/genetic-testing
**Foundation of the NLA: lipid panels**<br>Comprehensive information to help patients understand their lipid panel results.	https://www.learnyourlipids.com/heart-healthy-resources/lipid-panels/
**NAFCS scoring calculator**<br>Online calculators for the NAFCS scoring tool to help physicians distinguish FCS from other causes of severe HTG on the basis of clinical and laboratory tests.	https://www.lipid.org/nla/north-american-familial-chylomicronemia-calculator-or-nafcs-scoring-tool<br>https://tgaware.com/diagnosing-fcs/fcs-diagnostic-scoring/
**Diet and lifestyle**	
**Foundation of the NLA: low-fat cookbook for patients with FCS**<br>A collection of low-fat and flavorful recipes specifically designed for patients with FCS.	https://www.learnyourlipids.com/heart-healthy-resources/low-fat-cookbook/
**Action FCS: recipes**<br>A collection of low-fat recipes shared by members of the FCS community.	https://www.actionfcs.org/support/recipes/
**FCS nutrition and lifestyle**<br>A collection of FCS-friendly food plans and lifestyle recommendations.	https://www.knowyourtgs.com/fcs-nutrition-and-lifestyle/
**FDA-approved label**	
**Highlights of prescribing information**<br>Indications and usage instructions for olezarsen.	https://www.accessdata.fda.gov/drugsatfda_docs/label/2024/218614s000lbl.pdf
**EMA-approved label**	

**Summary of product characteristics**<br>Indications and usage instructions for olezarsen.	https://www.ema.europa.eu/en/medicines/human/EPAR/tryngolza#product-info

Reporting adverse events observed with olezarsen

Adverse events observed with olezarsen in practice should be reported to (800) 491-2664 or emailed to adverseevent@ionis.com. Suspected adverse reactions can also be reported to the FDA at 1-800-FDA-1088 or http://www.fda.gov/medwatch.

Contact details provided in the FDA-approved label should be consulted for the most up-to-date information.

All websites were last accessed on October 31, 2025. Information on patient access and support programs for olezarsen may be available online.

EMA, European Medicines Agency, FCS, familial chylomicronemia syndrome; FDA, US Food and Drug Administration; HTG, hypertriglyceridemia; NAFCS, North American Familial Chylomicronemia Syndrome; NLA, National Lipid Association.

Fig. S1. General usage instructions for olezarsen.

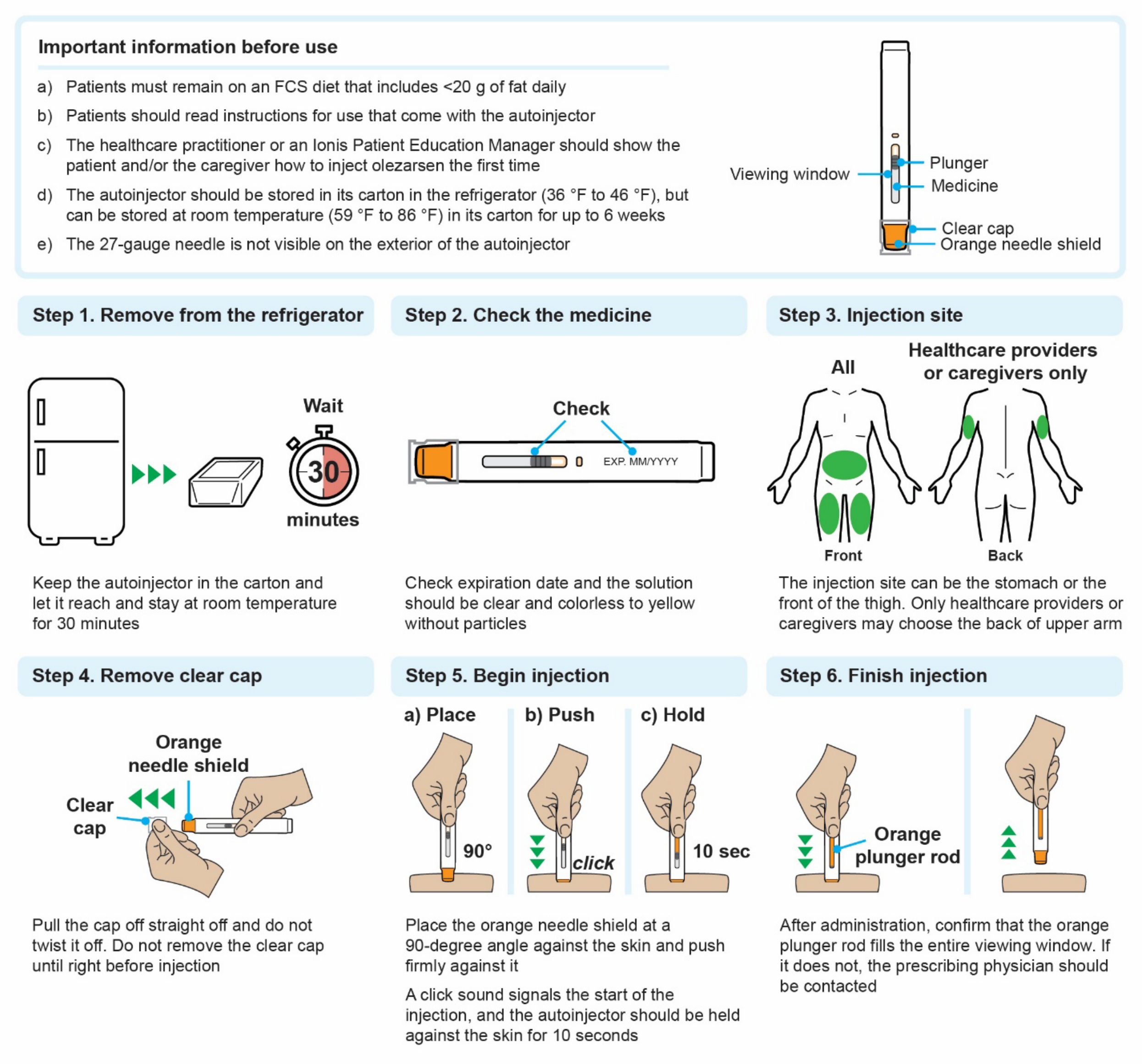

Comprehensive patient information and usage instructions for olezarsen are available in the FDA-approved prescribing information.

FCS, familial chylomicronemia syndrome; FDA, US Food and Drug Administration.

References

1. Moulin P, Dufour R, Averna M, Arca M, Cefalu AB, Noto D, et al. Identification and diagnosis of patients with familial chylomicronaemia syndrome (FCS): expert panel recommendations and proposal of an "FCS score". Atherosclerosis 2018;275:265-72.

2. Hegele RA, Ahmad Z, Ashraf A, Baldassarra A, Brown AS, Chait A, et al. Development and validation of clinical criteria to identify familial chylomicronemia syndrome (FCS) in North America. J Clin Lipidol 2024;19(1):83-94.
